# Algorithms in Allergy: Diagnosis and Treatment of Atopic Dermatitis Complicated by Eczema Herpeticum

**DOI:** 10.1111/all.16632

**Published:** 2025-07-02

**Authors:** Stephan Traidl, Annice Heratizadeh, Thomas Werfel

**Affiliations:** ^1^ Department of Dermatology and Allergy Hannover Medical School Hannover Germany; ^2^ Cluster of Excellence RESIST (EXC 2155), Hannover Medical School Hannover Germany

**Keywords:** atopic dermatitis, eczema herpeticum, HSV, type 2 immune response, virus

Eczema herpeticum (EH), first described by Moritz Kaposi in 1887 and therefore also known as Kaposi's varicelliform eruption, is a severe disseminated herpes simplex virus (HSV) infection primarily affecting individuals with atopic dermatitis (AD) [1]. EH can lead to significant morbidity and potentially life‐threatening complications such as encephalitis, pneumonia, and bacterial superinfection of the skin lesions. Early diagnosis and prompt management are critical. Although EH is considered a clinically relevant complication in patients with moderate‐to‐severe AD, robust global epidemiological data are lacking. Prevalence estimates are currently limited to national cohort studies, such as the TREATgermany registry, which reported a prevalence of EH in approximately 22% of patients with moderate‐to‐severe AD [2].

EH typically manifests with widespread, non‐grouped vesicular eruptions and/or erosions on eczematous or, more rarely, on unaffected skin of AD patients. Accompanying systemic symptoms often include fever, malaise, and lymphadenopathy. Secondary bacterial skin infections may further complicate the clinical picture.

Patients at highest risk are those with moderate‐to‐severe AD, which is also equivalent to insufficiently treated AD, and, particularly, patients with early onset of AD, allergic comorbidities, and/or a history of recurrent HSV infections [3]. Predisposing factors contributing to a disruption of skin barrier function include filaggrin loss‐of‐function mutations, single nucleotide polymorphisms (SNPs) in specific genes including, for instance, Claudin‐1, TSLP, and COL23A1, and type 2 immune polarization, which suppresses antiviral responses, notably interferon‐γ production [4, 5]. These are risk factors for increased susceptibility in a subset of AD patients culminating in the clinical presentation of recurrent EH, which is reported by 54.9% of EH patients [2]. Complications of EH include encephalitis, herpes keratitis, erythema multiforme, and HSV pneumonia and, as mentioned above, secondary bacterial infection of EH lesions. According to the current guidelines, awareness on both sides, the treating physicians and the patients, for example, supported by participation in therapeutic patient education programs [6], is a prerequisite for sufficient treatment for the earliest possible intervention to further prevent morbidity and improve outcomes in affected patients.

EH is primarily diagnosed clinically. Confirmatory diagnostics primarily include HSV polymerase chain reaction (PCR), which is the gold standard for HSV detection (Figure 1). Other diagnostic methods, such as histology and antigen detection via immunofluorescence, can be utilized if HSV PCR is unavailable. Clinical differential diagnoses include bacterial superinfection of eczema, impetigo, eczema coxsackium caused by coxsackie virus, and varicella zoster virus infections. While EH typically presents with monomorphic, non‐grouped vesicles on eczematous skin, eczema coxsackium often shows polymorphic vesiculobullous lesions, typically including acrofacial involvement optionally leading to acral desquamation. In unclear cases, PCR testing for both HSV and enteroviruses is recommended to distinguish between the two entities. Although impetigo may occasionally be associated with systemic symptoms, this is uncommon. Moreover, although initial skin lesions may include small superficial blisters, they typically lack the monomorphic vesicular eruption characteristic of EH. Varicella may also cause disseminated vesicular eruptions; however, the lesions are typically polymorphic, resembling Heubner's “celestial chart” and frequently involve the scalp and oral cavity, distinguishing it from the more monomorphic and localized presentation of eczema herpeticum.

**FIGURE 1:**
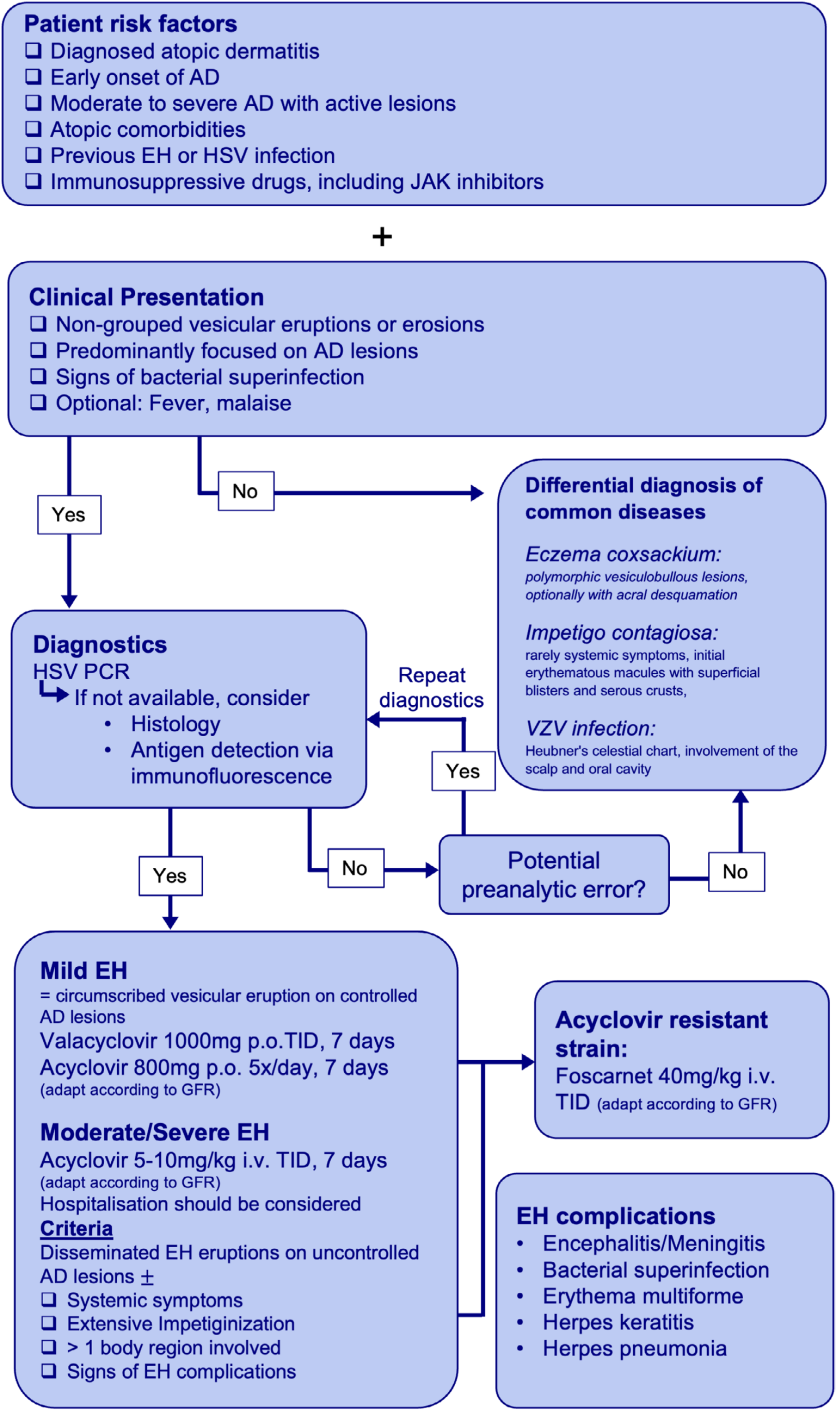
Diagnostic Criteria and Treatment Algorithm for Eczema herpeticum. AD, Atopic dermatitis; EH, Eczema herpeticum; GFR, Glomerular Filtration Rate; i.v., intravenous; JAK, Januskinase; p.o., per os; TID, Three times a day.

The management of EH involves both systemic antiviral and topical antiseptic treatment and further supportive care depending on the overall severity and complications. While mild EH can be defined as circumscribed vesicular eruptions on controlled AD lesions, moderate‐to‐severe EH would be seen as disseminated EH eruptions on uncontrolled AD lesions, potentially combined with further severity criteria such as presence at > 1 body region, clinical or laboratory signs for systemic inflammation such as fever, malaise, lymphadenopathy, or laboratory signs of organ dysfunction, and neurological symptoms including changes in consciousness, confusion, or neck stiffness indicating meningitis and encephalitis. These indicators also serve as criteria for hospitalization. An antiviral therapy for moderate/severe EH consists of intravenous acyclovir (5–10 mg/kg, three times daily) for 7 days, adjusted for renal function. For mild EH, oral valacyclovir (1 g, three times daily) or acyclovir applied in a dosage as used for the indication of herpes zoster are viable alternatives. Higher bioavailability of oral valacyclovir compared to oral acyclovir should be taken into account. Acyclovir‐resistant HSV strains can be treated intravenously with foscarnet (dosage for adults: 40 mg/kg TID [three times a day]). Additionally, novel drugs, such as pritelivir, a helicase‐primase inhibitor, are currently being tested in clinical trials, particularly for the treatment of acyclovir‐resistant HSV lesions. Adjunctive care includes treatment of secondary bacterial infections with topical disinfection treatment combined with systemic antibiotics when indicated. While topical corticosteroids in addition to valacyclovir have demonstrated potential faster healing in localized HSV infections in AD patients, there is currently no evidence supporting their efficacy in the management of EH [7]. While no specific follow‐up after successful treatment of an EH episode is suggested, patients should be thoroughly educated about the risk of recurrent EH and the importance of consistent and effective management of their underlying AD.

In patients with a history of EH the indication for systemic treatment for AD requires careful evaluation of risks and benefits (Figure 2). Dupilumab, an interleukin‐4 receptor antagonist, has proven efficacy in reducing EH risk by enhancing epidermal barrier integrity and counteracting type 2 inflammation [8, 9]. Whether this is also applicable to IL‐13 inhibitors (tralokinumab, lebrikizumab) has not yet been investigated. Therefore, the use of dupilumab (or other type 2 directed biologics) is preferred for biologic‐naïve individuals with moderate‐to‐severe AD and EH in the past, especially for those with recurrent EH. Conversely, JAK inhibitors (JAKi), including selective JAK1/2i, should be used with caution due to their potential to increase the risk for virus reactivation, particularly including HSV [10]. For EH‐high‐risk patients requiring JAK inhibitors or conventional systemic immunosuppression, a detailed assessment of recurrent HSV reactivation prior to treatment is essential. In case of recurrent HSV infections in the patient's history (≥ 3–6×/year), stand‐by antiviral therapies (e.g., valacyclovir 2 g BID [twice‐daily] for 1 day) can be considered as part of an integrated management plan. In case of insufficient HSV control, prophylactic treatment with valacyclovir 500 mg per day concomitantly to JAKi treatment over 6–12 months can be given in accordance the approved labeling [7, 11]. Close monitoring and thorough patient education are crucial to reducing recurrence and ensuring optimal outcomes.

**FIGURE 2:**
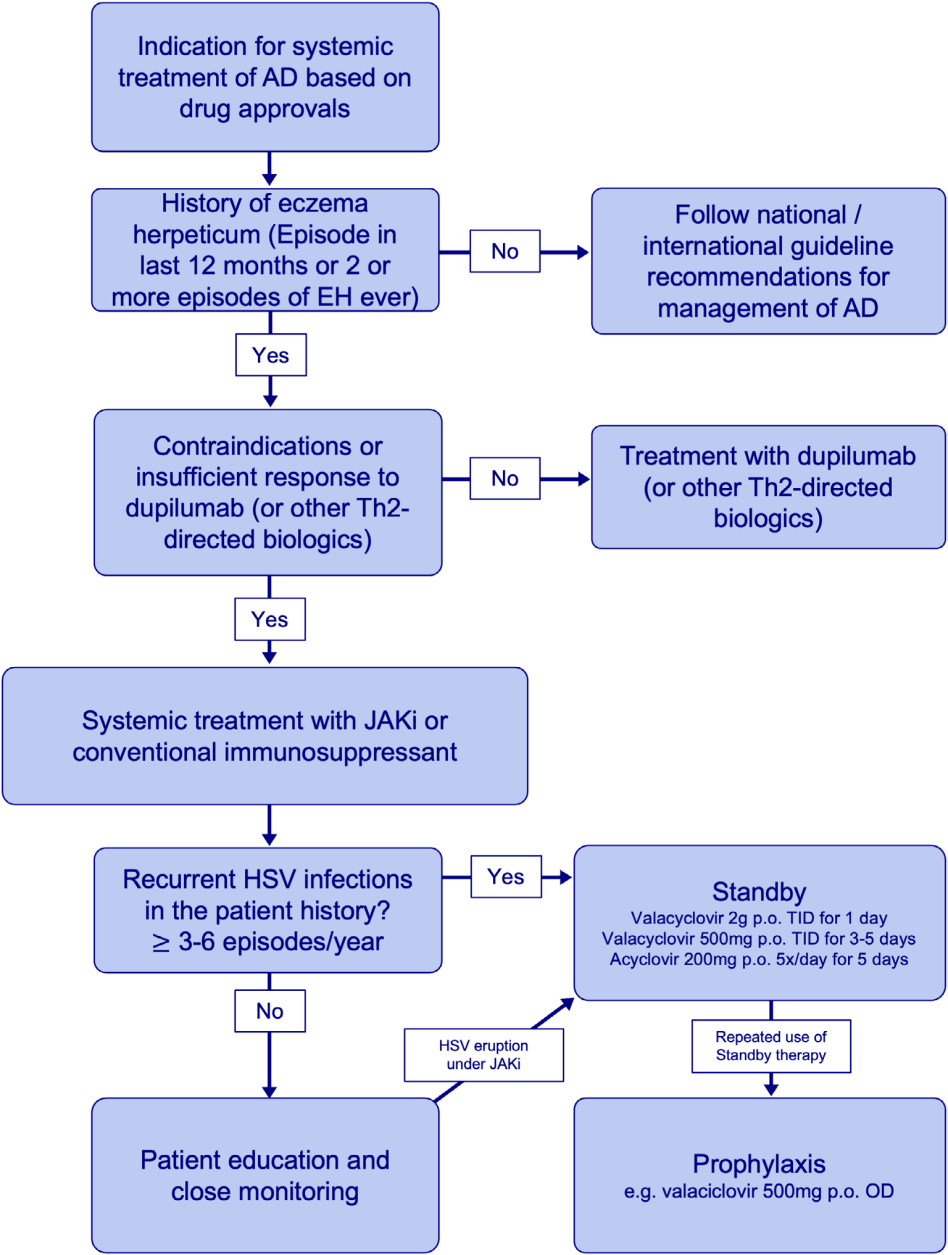
Systemic Treatment of patients with atopic dermatitis and a history of Eczema herpeticum (EH). AD, Atopic dermatitis; EH, Eczema herpeticum; JAKi, Januskinase inhibitor; OD, once daily; p.o., per os; TID, Three times a day.

EH represents a critical complication of AD, requiring rapid diagnosis and immediate intervention. Advances such as biologics like dupilumab offer significant potential to mitigate EH risk in predisposed AD populations. Further research should focus on identifying biomarkers for high‐risk individuals that would profit from preventive, targeted therapy.

## Conflicts of Interest

S.T. received research grants from Sanofi and Novartis Foundation. He served as a consultant and lecturer for AbbVie, ALK, Galderma, Incyte, Lilly Pharma, Leo Pharma, Janssen, Novartis, and Sanofi. A.H. declares consulting fees and/or travel grants from Janssen, AbbVie, Almirall, Lilly, Novartis, Pierre Fabre, Sanofi, Beiersdorf, Leo Pharma, Nutricia, Hans Karrer, Meda, Klinge Pharma, ALK, and Pfizer.

## Data Availability

Data sharing is not applicable to this article as no new data were created or analyzed in this study.
